# Supplementary material for: FDRestimation: Flexible False Discovery Rate Computation in R
Source: F1000Res. 2021 Oct 19;10:441. Originally published 2021 Jun 3. [Version 2] doi: 10.12688/f1000research.52999.2 (PMC8669776; doi:10.12688/f1000research.52999.2)
Supplement: Peer review history

# Peer review: Reviewer response for version 2

**DOI:** 10.5256/f1000research.78269.r101444

**Published:** 2021-12-08

**Authors:** David Colquhoun

**Affiliations:** 1 Department of Neuroscience, Physiology and Pharmacology, University College London, London, UK

First, I must say that my knowledge about methods used for multiple comparisons is not great. That being said, I find the paper entirely convincing.

 My comments will be restricted to matters of clarity and notation, apart from one technical point. 

 I must confess to having played a small part in the confusion about notation. In 2014 I wrote a paper about FDR but I was talking about the interpretation of p values from single tests, not multiple comparisons ^1^. I said in that paper "IR should clarify that this paper is not about multiple comparisons. It is well known that high false discovery rates occur when many outcomes of a single intervention are tested." This was a mistake because up to that point, the term FDR had been used almost exclusively for the correction of p values for multiple comparisons. 

 I therefore added to the confusion by changing my notation from FDR to False positive risk (FPR) in my 2017 ^2^ and 2019 ^3^ papers.

 In the 2017 paper, I wrote

 "It is worth noting that all the methods for correcting for multiple comparisons aim to correct only the type 1 error. The result is, therefore, a (corrected) *p-*value, so it will still underestimate the false positive risk, for the reasons to be described." ^2^

 I was therefor very happy to see in your paper the section labelled "Lower bound on the FDR". There you use a lower bound which you attribute to Berger (1985) - a reference that is missing from the reference list. Your eqn 7 is the same as eqn 1.2 in Berger & Sellke JASA,1987), which they attribute to, Edwards, Lindeman and Savage (1963).

 In my 2019 paper ^3^, I use a similar approach (which I attribute to Goodman,1999b). I compare it with 2 other methods in Fig. 3 of that paper. As you can see, it agrees will with my own approach which differs only by using the *t* distribution rather than normal. But in order to get this agreement it is necessary to multiply by 2 the exponential term in the likelihood ratio exp(−z^2/2) (see appendix A3, eqn A9). It's pointed out in Held and Ott,2018,eq.13 that this factor of two is necessary if one is talking about 2-tailed tests. Since you are, perhaps you should add it. This is confirmed by the fact that when I run your p.fdr with a single p value of 0;05 it gives what you call FDR (and I call FPR_50) as 0.128, whereas with the factor of 2 included I get FPR of 0.227 corresponding to a likelihood ration of 3.4 -and it's a well-known result that p=0.05 gives a likelihood ratio of around 3 by a variety of arguments.

 In summary, I think that the paper would be improved by two things.

1. A discussion of the nomenclature used in this area. Is it sensible to use FDR only for corrected p values, or should the same term be used when you are referring to P(H0 | data)? I'd argue that FPR is more appropriate and that "risk" is more appropriate than "rate", given that the likelihood ratio is calculated by the p-equals approach, so we are considering only evidence from the one experiment m that we're analysing rather than long term error rates.
2. Inclusion of the factor of 2 in the calculation of the likelihood ratio (or at least a mention of the fact that your expression is not right for 2 tail tests).

Are the conclusions about the tool and its performance adequately supported by the findings presented in the article?

Yes

Is the rationale for developing the new software tool clearly explained?

Yes

Is the description of the software tool technically sound?

Yes

Are sufficient details of the code, methods and analysis (if applicable) provided to allow replication of the software development and its use by others?

Yes

Is sufficient information provided to allow interpretation of the expected output datasets and any results generated using the tool?

Yes

Reviewer Expertise:

I am not a professional statistician. I'm a biophysicist with a strong interest in statistics. In 1971 I wrote a textbook, Lectures on Biostatistics, and have recently written three papers about the (mis)interpretation of p values

I confirm that I have read this submission and believe that I have an appropriate level of expertise to confirm that it is of an acceptable scientific standard.

## References

[1] : An investigation of the false discovery rate and the misinterpretation of p-values. *R Soc Open Sci* .2014;1(3) : 10.1098/rsos.140216 140216 10.1098/rsos.140216 26064558PMC4448847

[2] : A more principled use of the p-value? Not so fast: a critique of Colquhoun's argument. *R Soc Open Sci* .2019;6(5) : 10.1098/rsos.181519 181519 10.1098/rsos.181519 31218019PMC6549987

[3] : The False Positive Risk: A Proposal Concerning What to Do Aboutp -Values. *The American Statistician* .2019;73(sup1) : 10.1080/00031305.2018.1529622 192-201 10.1080/00031305.2018.1529622

# Peer review: Reviewer response for version 2

**DOI:** 10.5256/f1000research.78269.r97323

**Published:** 2021-11-08

**Authors:** Ted Westling, Yujian Wu

**Affiliations:** 1 Department of Mathematics and Statistics, University of Massachusetts Amherst, Amherst, MA, USA

The authors have clearly answered all the questions, updated the figures and code as suggested.

Are the conclusions about the tool and its performance adequately supported by the findings presented in the article?

Yes

Is the rationale for developing the new software tool clearly explained?

Yes

Is the description of the software tool technically sound?

Yes

Are sufficient details of the code, methods and analysis (if applicable) provided to allow replication of the software development and its use by others?

Partly

Is sufficient information provided to allow interpretation of the expected output datasets and any results generated using the tool?

Partly

Reviewer Expertise:

Non-parametric estimation and inferrence

We confirm that we have read this submission and believe that we have an appropriate level of expertise to confirm that it is of an acceptable scientific standard.

# Peer review: Reviewer response for version 1

**DOI:** 10.5256/f1000research.56339.r86958

**Published:** 2021-07-07

**Authors:** Ted Westling, Yujian Wu

**Affiliations:** 1 Department of Mathematics and Statistics, University of Massachusetts Amherst, Amherst, MA, USA

This article emphasizes the necessity of distinguishing between the computation of adjusted p-values for FDR control and FDR estimation. A variety of commonly used methods for FDR estimation are introduced in the paper and the implemented package makes the methods easily accessible. The authors also provide a new method for estimation of the proportion for the null hypothesis. A simulation study verifies the new method works well when compared with other existing methods. The authors also have detailed interpretations of the functions in the package, together with some example code in the following section. Overall, the paper develops in a logical way with replicable results.

 We have several comments and questions about the article and package:

 1. All three functions have one common argument, ”z values”, which can either be a vector or a string. When we tried to pass only one vector to the argument (without providing p-values) to compute the FDR, the function didn’t work. we had to pass the p-values corresponding to the z-values to make it work. Given that there’s a z-value based approach for FDR estimation, is it possible to make the function work whenever it takes a numerical vector for the argument ”z values”?

 For example, if we run the following code:

 ```

 z <- runif(10)

 p.fdr(zvalues = z)

 ```

 It will give me an error message "Error in TRUE %in% (pvalues > 1 | pvalues < 0) : argument "pvalues" is missing, with no default", unless we run it this way:

 ```

 p.fdr(pvalues = pnorm(z))

 ```

 2. The lower bound on FDR is introduced in the paper as one way of identifying optimistic FDR estimations. Is it reasonable to incorporate this lower bound into the output of the function ”p.fdr”?

 3. In terms of algorithm 1, is it necessary to store the heights for every bin? Is the height of the last bin alone enough?

 4. In figure 6, when the true null proportion is 1, in which case the null distribution has a uniform distribution, ${\hat{\pi }}_{0}$ seems to be biased downward. Shouldn’t the histogram reflect the uniform distribution better than other skewed distributions (when the proportion is less than 1), thus a better estimation of *π*_0_?

 5. Though it is claimed that the last histogram height method has the minimum MSE, Figure 6 only shows the bias of the estimators. It would be better if there’s another figure showing the variance and/or MSE for all estimators.

 6. Please add axis labels and interpretable legends to every figure.

 7. The default method when there are ties in the ranks is important. The authors set the default to “random”, which has the downside that the results may differ on the repeated running of the same code. Can the authors discuss this choice? In addition, we think if there are ties, the functions should provide a warning or message about the presence of ties and how they are being handled.

Are the conclusions about the tool and its performance adequately supported by the findings presented in the article?

Yes

Is the rationale for developing the new software tool clearly explained?

Yes

Is the description of the software tool technically sound?

Yes

Are sufficient details of the code, methods and analysis (if applicable) provided to allow replication of the software development and its use by others?

Partly

Is sufficient information provided to allow interpretation of the expected output datasets and any results generated using the tool?

Partly

Reviewer Expertise:

Non-parametric efficient estimation

We confirm that we have read this submission and believe that we have an appropriate level of expertise to confirm that it is of an acceptable scientific standard, however we have significant reservations, as outlined above.

# Response

**Published:** 2021-10-06

**Authors:** Megan Murray

**Affiliations:** Vanderbilt University Medical Center, USA

Thank you very much for your time and suggestions. We have incorporated those suggestions and revised accordingly, which resulted in an improved paper. Some key issues were noted, and we clarify those below.

1. This is a subtle but important issue. The false discovery rates and adjusted p-values are all computed using the provided ‘pvalues’ vector. The z-values are used only for graphing in the z-value space and for computing the lower bound on the FDR. Remember that the z-values alone do not uniquely determine the p-value; it matters if the p-values are two-sided, one-sided in either direction, or adjusted for continuity or multiple looks. Accordingly, we found it simpler to require the p-values instead of hard-coding certain assumptions. We added a warning message for when the ‘pvalues’ vector is missing. It reads: “Error: The ‘pvalues’ vector is required for all FDR computations.”
2. This is already included in the function. The lower bound is applied in all cases where the user has only supplied a single p-value. We have changed the class name of the output for this situation to improve user clarity.
3. Perhaps. However, we call the graphics package function “hist()”, which automatically computes and stores every bin height. So, changing this would not improve the run time. However, the all the bin heights do not have to be stored for this method.
4. When the true null proportion is one, all of methods produce estimates that are biased downward (largely because the estimates are capped at 1). Accordingly, there is no unbiased estimate of the null proportion. Moreover here “better” is open for debate, as there is a bias-variance trade-off among the methods.
5. We have included the plots of the MSE in new Figure 7 so readers can assess for themselves the relative improvements. In our opinion, ‘Last Histogram Height’ and ‘Storey’ methods were the two methods that performed best over the widest range of cases. 
6. We have submitted an updated version with a few axis labels and interpretable legends added. For Figures 1,2,4,8, and 9 we do not label the y-axis label to avoid confusion between the adjusted p-values and raw p-values (the y-axis space is the unit interval).
7. Thank for your input here. We added the suggested warning to alert the user when ties are present. We chose the default “random” because in most cases the order of the p-values vector is not contextually meaningful. To address the reproducibility issue, the user can set a seed before running the function and reproduce the exact results.
